# Evaluation of Coronary Artery Luminal Diameters in Patients with Pulmonary Arterial Hypertension

**DOI:** 10.3390/medicina61030381

**Published:** 2025-02-22

**Authors:** Ufuk Yildirim, Gulten Taskin, Meliyke Hatun Baser, Burak Tugmen, Busranur Yaliniz, Ilkay Camlidag, Murat Meric

**Affiliations:** 1 Department of Cardiology, Faculty of Medicine, Ondokuz Mayis University, 55200 Samsun, Turkey; meliykehatun@gmail.com (M.H.B.); drmeric@hotmail.com (M.M.); 2 Department of Radiology, Faculty of Medicine, Ondokuz Mayis University, 55200 Samsun, Turkey; gultenolmez@gmail.com (G.T.); ilkayozaydin@hotmail.com (I.C.); 3 Faculty of Medicine, Ondokuz Mayis University, 55200 Samsun, Turkey; buraktugmen@outlook.com (B.T.); busrayaliniz1@gmail.com (B.Y.)

**Keywords:** coronary artery luminal diameter, pulmonary arterial hypertension, cardiac computed tomography angiography

## Abstract

*Background and Objectives*: Recent studies have demonstrated that pulmonary arterial hypertension (PAH) is a vascular disease that extends beyond the pulmonary vasculature. PAH has been associated with increased intramural coronary arteriolar medial thickness and decreased coronary arteriolar luminal area in both human and experimental models of the disease. The objective of this study was to assess the luminal diameter of epicardial coronary arteries in patients with PAH. *Materials and Methods*: Fifty patients with PAH who underwent cardiac computed tomography (CT) angiography at our center were included in this retrospective study. Fifty patients without pulmonary hypertension matched for age, sex, and coronary dominance were also included. Coronary artery luminal diameters measured by cardiac CT angiography were compared between the groups, in addition to baseline characteristics and standard cardiac measurements. Correlation analysis was also performed. *Results*: The diameters of the left main coronary artery, left anterior descending artery, and left circumflex artery were comparable between the groups. However, the right coronary artery (RCA) diameter was found to be greater in the PAH group (3.51 ± 0.66 mm vs. 3.02 ± 0.49 mm, *p* < 0.001). The RCA diameter exhibited a positive moderate correlation with the main pulmonary artery diameter (R = 0.517, *p* < 0.001), right atrial area (R = 0.515, *p* < 0.001), and right ventricular diastolic diameter (R = 0.506, *p* < 0.001). *Conclusion*: PAH may be associated with an increase in the RCA diameter.

## 1. Introduction

Pulmonary arterial hypertension (PAH) is a devastating disease characterized by progressive remodeling of the distal pulmonary arteries. Recent studies have demonstrated that PAH is a vascular disease that extends beyond the pulmonary vasculature. Reduced cerebral blood flow [1], impaired brachial flow-mediated vasodilation [2], increased urinary albumin excretion as a possible early marker of renal endothelial dysfunction [3], abnormal episcleral vessels [4], decreased nailfold capillary density [5], and lower sublingual blood flow index [6] are among the extrapulmonary findings of PAH that may be suggestive of an underlying systemic vascular abnormality. PAH has also been associated with increased intramural coronary arteriolar medial thickness and decreased coronary arteriolar luminal area in both human and experimental models of the disease [7,8]. Computed tomography (CT) imaging has traditionally been used in the evaluation of patients with PAH. A combined pulmonary and cardiac CT angiography provides valuable information on the pulmonary vasculature and PAH etiology, as well as cardiac morphology and coronary arterial anatomy [9]. The objective of this study was to assess the luminal diameter of epicardial coronary arteries in patients with PAH undergoing cardiac CT angiography.

## 2. Materials and Methods

In this retrospective study, two groups of patients were enrolled. The PAH group comprised 50 patients diagnosed with group 1 pulmonary hypertension (PH) according to the clinical classification of PH who underwent cardiac CT angiography at our center between August 2017 and August 2024. PH was defined by a mean pulmonary arterial pressure greater than 20 mmHg at rest, as measured by right heart catheterization (RHC) [10]. The control group was selected from patients without PH who underwent cardiac CT angiography at our center between August 2023 and August 2024, and 50 patients who were matched with the PAH group for age, sex, and coronary dominance were included. The exclusion criteria were as follows: PH classes other than group 1, age less than 18 years, diabetes, hypercholesterolemia defined as total cholesterol higher than 240 mg/dL at any time [11], chronic kidney disease defined as glomerular filtration rate lower than 60 mL/min/1.73 m^2^ for at least 3 months [12], anemia defined as hemoglobin level less than 12.0 g/dL in women and 13.0 g/dL in men, chronic obstructive pulmonary disease, previous myocardial infarction, previous percutaneous coronary intervention, previous coronary artery bypass grafting, left ventricular (LV) ejection fraction less than 50%, more than mild pericardial effusion, external compression of the left main coronary artery (LM), and any evidence of atherosclerotic disease in cardiac CT angiography. The study was conducted in accordance with the Declaration of Helsinki, and the protocol was approved by the institutional ethics committee.

The baseline characteristics of the study population were recorded from the hospital database and the National Health Record System. Age, sex, history of hypertension, and body mass index were noted. Individuals who had smoked at least 100 cigarettes during their lifetime were classified as smokers [13]. The Du Bois formula was employed to estimate the body surface area (BSA) [14].

All CT examinations were performed with a 64-slice multi-detector CT scanner (Discovery CT750 HD, General Electric Healthcare, Waukesha, WI, USA) using a retrospective electrocardiography-gated acquisition protocol. Patients were positioned supine, feet first in the gantry. Scanning was initiated using the bolus tracking technique with a region of interest (ROI) placed in the ascending aorta. A 50–90 mL weight-based dose of nonionic iodinated intravenous contrast agent was administered via a power injector at a rate of 3–5 mL/s, followed by a 30 mL saline bolus. Image acquisition was initiated when ROI enhancement reached 300 HU. The acquisition parameters were as follows: gantry rotation time 0.35 s, detector coverage 40 mm, slice thickness 2.5 mm, slice interval 0.625 mm, and pitch 0.2:1. The images were reconstructed at 6 different intervals with 10% increments across the R-R cycle (45–95%).

**Figure 1:**
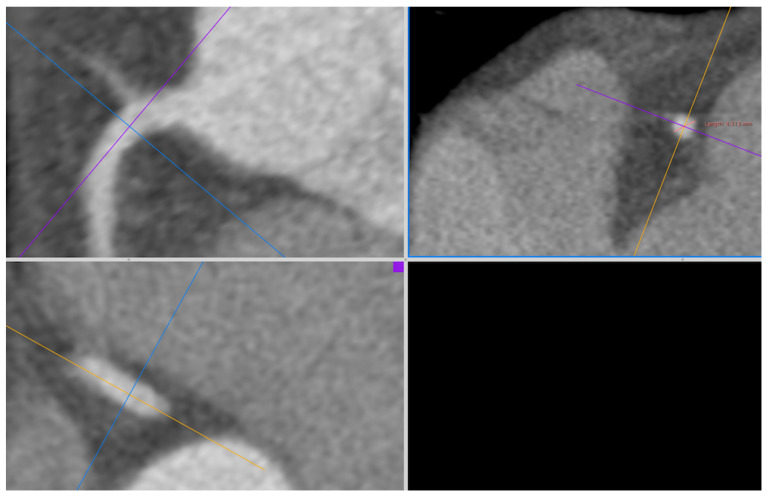
Three-dimensional curved MPR CT image depicting the measurement of RCA diameter; CT: computed tomography; MPR: multiplanar reformatted; RCA: right coronary artery.

The CT examinations were analyzed by a radiologist with 10 years of experience in cardiac imaging. All assessments were executed using Horus software version 3.3.6. The measurement of coronary artery diameters, ascending aortic diameter (AscAoD), and main pulmonary artery diameter (MPAD) was conducted on three-dimensional curved multiplanar reformatted images. The measurement of coronary artery diameters and AscAoD was conducted from oblique axial planes that were perpendicular to the vessel axis on coronal and sagittal images. The luminal diameter of the LM was measured from the midsection, and the luminal diameters of the left anterior descending artery (LAD), left circumflex artery (LCX), and right coronary artery (RCA) were measured within 1 cm of the origin of each artery (Figure 1). The AscAoD measurement was taken at a point approximately 4 cm above the aortic annulus. The MPAD measurement was taken from the widest part before the bifurcation, where it was perpendicular to the vessel wall. The measurement of left atrial area (LAA) and right atrial (RA) area (RAA) was performed on four-chamber views by tracing the inner borders of the chambers at the end-systolic phase on the slices where the chambers appeared the largest. The measurement of LV diastolic diameter (LVDD) and right ventricular (RV) diastolic diameter (RVDD) was performed on four-chamber views from the midseptum to the lateral wall at the end-diastolic phase on the slices where the chambers appeared the largest. The septal thickness (ST) was measured in the midseptum on end-diastolic short-axis views, and the posterior wall thickness (PWT) was measured from the basal inferior wall on end-diastolic short-axis views. The LV mass index (LVMi) was calculated by indexing the LV mass estimated by Devereux’s formula to BSA [15].

The variability of coronary artery luminal diameters was assessed by re-examining the CT images of 10 patients selected at random, with 5 subjects from each group. The measurements were repeated by the same radiologist after at least 1 week for the purpose of assessing intraobserver variability, and by a different radiologist for the purpose of assessing interobserver variability.

The analysis of the research data was conducted using the Statistical Package for the Social Sciences version 25. Descriptive statistics for categorical variables are presented in terms of frequencies and percentages. The chi-squared test was employed to compare categorical variables. The distribution of numerical variables was determined using the Kolmogorov–Smirnov test. Descriptive statistics for numerical variables exhibiting a normal distribution are expressed as mean ± standard deviation. The independent samples *t*-test was employed to compare these variables. Descriptive statistics for numerical variables without a normal distribution are given as median (minimum–maximum). The Mann–Whitney U test was employed to compare these variables. Pearson’s correlation coefficient was utilized to reveal the magnitude and direction of the relationships between numerical variables exhibiting a normal distribution, while Spearman’s correlation coefficient was applied for numerical variables without a normal distribution. The strength of a relationship was categorized as follows: strong if R ≥ 0.7, moderate if 0.5 ≤ R < 0.7, and weak if 0.3 ≤ R < 0.5. The variability of coronary artery luminal diameters was evaluated using the intraclass correlation coefficient (ICC) by Cronbach. The statistical significance level was set at *p* ≤ 0.05.

## 3. Results

The groups were similar in terms of the baseline characteristics (Table 1). Of the 50 patients in the PAH group, 6 (12%) had idiopathic PAH, 2 (4%) had PAH associated with portal hypertension, and 42 (84%) had PAH associated with congenital heart disease (CHD).

The ST, PWT, and AscAoD were comparable between the groups. The LVDD and LVMi were significantly lower in the PAH group (*p* < 0.001 and *p* = 0.031, respectively). The LAA, RAA, RVDD, and MPAD were found to be significantly higher in the PAH group (*p* = 0.001, *p* < 0.001, *p* < 0.001, and *p* < 0.001, respectively) (Table 2).

Right coronary dominance was present in 47 of the 50 patients in both groups. The diameters of the LM, LAD, and LCX were comparable between the groups. However, the RCA diameter was found to be greater in the PAH group (*p* < 0.001) (Table 3).

Correlation analysis revealed no strong relationship between coronary artery luminal diameters and standard cardiac measurements. The RCA diameter exhibited a positive moderate correlation with the MPAD (R = 0.517, *p* < 0.001), RAA (R = 0.515, *p* < 0.001), and RVDD (R = 0.506, *p* < 0.001). Additionally, a positive moderate correlation was identified between the LM diameter and the PWT (R = 0.507, *p* < 0.001). Other significant but weak relationships were also found between coronary artery luminal diameters and standard cardiac measurements (Table 4).

The ICC analysis for the variability of coronary artery luminal diameters is presented in Table 5.

## 4. Discussion

This study revealed that the PAH group had a larger RCA diameter, which exhibited a positive moderate correlation with the MPAD, RAA, and RVDD. The mean RCA diameter was 3.51 mm in the PAH group and 3.02 mm in the control group. Despite the considerable statistical significance, a 0.49 mm difference in vessel diameter might be perceived as inconsequential. However, the CT device utilized in the present study had a spatial resolution of 0.23 mm. Therefore, even after accounting for interobserver variability, the observed difference in vessel diameter was meaningful. To our knowledge, this is the first study to demonstrate increased RCA diameter in patients with PAH.

Coronary arterial remodeling associated with PAH has garnered interest from researchers in recent years. Akhavein et al. reported increased intramural coronary arteriolar medial thickness and decreased coronary arteriolar luminal area throughout the RV free wall, interventricular septum, and LV posterior wall in rats with monocrotaline (MCT)-induced PAH. However, the authors suggested that this result might be attributable to the direct toxic effects of MCT on the myocardium, as the findings were independent of the severity of PH [8]. In a study including only three patients with PAH, Meloche et al. observed an increase in coronary arteriolar wall thickness in the postmortem histopathological examination of these patients compared to the control group. They also observed an increase in coronary arteriolar wall thickness and a decrease in coronary perfusion in rat models of MCT-induced PAH as well as Sugen/hypoxia-induced PAH. Furthermore, the authors reported that these findings were similar in both ventricles [7]. Sun et al. demonstrated that endothelium-dependent vasodilation of the coronary microcirculation was impaired in rats with MCT-induced PAH, with the most pronounced impairment in the RCA [16]. However, it is imperative to acknowledge the limitations of MCT-induced PAH as a representation of clinical conditions.

In the present study, which was designed to evaluate the luminal diameter of epicardial coronary arteries, the RCA diameter was found to be larger in the PAH group. A number of factors have been shown to influence coronary artery luminal diameters, one of which is flow demand [17]. Increased RV afterload in patients with PAH results in RV hypertrophy, which leads to increased flow demand [18]. The RCA supplies blood primarily to the RV and RA. Enlargement of the RV and RA has been well described in patients with PAH, and was also evident in our study. Moreover, the RCA diameter had a positive moderate relationship with the RVDD, RAA, and also MPAD in our study. The larger RCA diameter in the PAH group may represent an adaptive mechanism to meet the increased flow demand. RV hypertrophy in PAH is accompanied by thickening of the wall of myocardial arterioles and reduction in their luminal diameter, which inevitably causes a decrease in the efficiency of blood supply to the RV myocardium at the level of microcirculatory vessels [7,8]. Arterioles are compelled to augment the thickness of the media and diminish the diameter of their lumen to counteract the increased external pressure from the myocardium during RV systole to ensure the minimum required level of blood flow. A decrease in the luminal diameter of arterioles leads to deterioration of perfusion of the hypertrophied RV myocardium in PAH. The increase in the luminal diameter of the epicardial segment of the RCA may be a consequence of its passive dilation due to elevated resistance in myocardial arterioles. The larger RCA observed in the PAH group of our study can be attributed to these potential mechanisms.

Another positive moderate relationship was found between the LM diameter and the PWT in the present study. This finding is consistent with the literature. Dodge et al. demonstrated that coronary artery luminal diameter was increased in men with LV hypertrophy or enlargement. The authors suggested that the increase in coronary artery luminal diameter might be an adaptive phenomenon that emerges to meet the increased flow demand of the myocardium [17].

In this study, additional alterations beyond the larger RCA diameter were detected in the PAH group. Increased RAA, RVDD, and MPAD and decreased LVDD, which were observed in our study, are well-known structural alterations in patients with PAH. Reduced LVMi was most likely associated with reduced LVDD. The PAH group had an increased LAA, even though these patients had normal pulmonary arterial wedge pressure measured by RHC. Although not statistically significant, there was a tendency for smaller AscAoD and LCX diameter in the PAH group. Further research is clearly needed to clarify these findings.

The present study was subject to several limitations. Firstly, the sample size was relatively small. Secondly, given that the majority of patients in the PAH group had PAH associated with CHD, the results may not be applicable to all PAH subgroups. Finally, we did not have data on concomitant medication use, which might have influenced the results.

## 5. Conclusions

PAH may be associated with an increase in the RCA diameter. Subsequent investigation employing modalities that have the potential to provide more insights, such as coronary intravascular ultrasound, may facilitate a more comprehensive understanding of the subject.

## Figures and Tables

**Table 1:** Baseline characteristics of the study population.

Variable	PAH Group (n = 50)	Control Group (n = 50)	*p*
Age (year)	46.9 ± 14.7	47.0 ± 14.5	0.984
Female (% *)	38 (76.0)	38 (76.0)	1.000
Smoking (% *)	6 (12.0)	9 (18.0)	0.401
Hypertension (% *)	9 (18.0)	10 (20.0)	0.799
Body mass index (kg/m^2^)	27.46 ± 5.08	28.45 ± 4.76	0.314
Body surface area (m^2^)	1.813 ± 0.191	1.858 ± 0.189	0.246

PAH: pulmonary arterial hypertension. * Column percentage.

**Table 2:** Standard cardiac measurements.

Variable	PAH Group (n = 50)	Control Group (n = 50)	*p*
LVDD (mm)	39.4 ± 4.7	45.1 ± 4.6	<0.001
ST (mm)	9.4 ± 1.6	9.0 ± 1.4	0.195
PWT (mm)	8.0 ± 1.3	7.9 ± 1.2	0.798
LVMi (g/m^2^)	58.1 ± 17.9	62.6 ± 15.9	0.031
LAA (cm^2^)	23.4 ± 7.8	19.0 ± 3.7	0.001
RAA (cm^2^)	31 (18–62)	17 (12–27)	<0.001
RVDD (mm)	53.2 ± 7.0	37.7 ± 4.3	<0.001
MPAD (mm)	33.9 ± 6.2	24.0 ± 3.9	<0.001
AscAoD (mm)	29.6 ± 5.5	31.9 ± 6.2	0.074

AscAoD: ascending aortic diameter; LAA: left atrial area; LVDD: left ventricular diastolic diameter; LVMi: left ventricular mass index; MPAD: main pulmonary artery diameter; PAH: pulmonary arterial hypertension; PWT: posterior wall thickness; RAA: right atrial area; RVDD: right ventricular diastolic diameter; ST: septal thickness.

**Table 3:** Coronary artery luminal diameters.

Variable	PAH Group (n = 50)	Control Group (n = 50)	*p*
LM diameter (mm)	4.20 ± 0.74	4.15 ± 0.64	0.574
LAD diameter (mm)	3.49 ± 0.55	3.48 ± 0.60	0.931
LCX diameter (mm)	2.52 ± 0.53	2.72 ± 0.54	0.065
RCA diameter (mm)	3.51 ± 0.66	3.02 ± 0.49	<0.001
Right coronary dominance (% *)	47 (94.0)	47 (94.0)	1.000

LAD: left anterior descending artery; LCX: left circumflex artery; LM: left main coronary artery; PAH: pulmonary arterial hypertension; RCA: right coronary artery. * Column percentage.

**Table 4:** Correlation analysis.

Variable	LM Diameter		LAD Diameter		LCX Diameter		RCA Diameter	
	R	*p*	R	*p*	R	*p*	R	*p*
LVDD	0.145	0.151	0.047	0.641	0.136	0.177	−0.061	0.549
ST	0.406	<0.001	0.264	0.008	0.270	0.007	0.359	<0.001
PWT	0.507	<0.001	0.339	0.001	0.348	<0.001	0.336	0.001
LVMi	0.330	0.001	0.166	0.099	0.168	0.094	0.154	0.126
LAA	0.312	0.002	0.333	0.001	0.245	0.014	0.421	<0.001
RAA	0.241	0.016	0.193	0.054	−0.001	0.993	0.515	<0.001
RVDD	0.284	0.004	0.135	0.179	0.022	0.828	0.506	<0.001
MPAD	0.347	<0.001	0.304	0.002	0.090	0.375	0.517	<0.001
AscAoD	0.332	0.001	0.398	<0.001	0.233	0.020	0.210	0.036

AscAoD: ascending aortic diameter; LAA: left atrial area; LAD: left anterior descending artery; LCX: left circumflex artery; LM: left main coronary artery; LVDD: left ventricular diastolic diameter; LVMi: left ventricular mass index; MPAD: main pulmonary artery diameter; PWT: posterior wall thickness; RAA: right atrial area; RCA: right coronary artery; RVDD: right ventricular diastolic diameter; ST: septal thickness.

**Table 5:** Intraobserver and interobserver variability.

Variable	Intraobserver Variation			Interobserver Variation		
	ICC	95% CI	*p*	ICC	95% CI	*p*
LM diameter	0.972	0.886–0.993	<0.001	0.945	0.779–0.986	<0.001
LAD diameter	0.943	0.772–0.986	<0.001	0.910	0.636–0.978	0.001
LCX diameter	0.966	0.865–0.992	<0.001	0.912	0.647–0.978	0.001
RCA diameter	0.959	0.836–0.990	<0.001	0.979	0.916–0.995	<0.001

CI: confidence interval; ICC: intraclass correlation coefficient; LAD: left anterior descending artery; LCX: left circumflex artery; LM: left main coronary artery; RCA: right coronary artery.

## Data Availability

The datasets generated and analyzed during the current study are available from the corresponding author upon reasonable request. Access to the data is granted to researchers who are interested in replicating or further investigating the findings of this study, in accordance with applicable ethical guidelines and privacy regulations.
